# Advances in the Diagnosis and Treatment of Krabbe Disease

**DOI:** 10.3390/ijns7030057

**Published:** 2021-08-18

**Authors:** David A Wenger, Paola Luzi, Mohammad A. Rafi

**Affiliations:** Lysosomal Diseases Testing Laboratory, Department of Neurology, Sidney Kimmel College of Medicine at Thomas Jefferson University, Philadelphia, PA 19107, USA; paola.luzi@jefferson.edu (P.L.); mohammad.rafi@jefferson.edu (M.A.R.)

**Keywords:** Krabbe disease, galactocerebrosidase, GALC gene, diagnosis, treatment, newborn screening, animal models

## Abstract

Krabbe disease is an autosomal recessive leukodystrophy caused by pathogenic variants in the galactocerebrosidase (GALC) gene. GALC activity is needed for the lysosomal hydrolysis of galactosylceramide, an important component of myelin. While most patients are infants, older patients are also diagnosed. Starting in 1970, a diagnosis could be made by measuring GALC activity in leukocytes and cultured cells. After the purification of GALC in 1993, the cDNA and genes were cloned. Over 260 disease-causing variants as well as activity lowering benign variants have been identified. While some pathogenic variants can be considered “severe,” others can be considered “mild.” The combination of alleles determines the type of Krabbe disease a person will have. To identify patients earlier, newborn screening (NBS) has been implemented in several states. Low GALC activity in this screening test may indicate a diagnosis of Krabbe disease. Second tier testing as well as neuro-diagnostic studies may be required to identify those individuals needing immediate treatment. Treatment of pre-symptomatic or mildly symptomatic patients at this time is limited to hematopoietic stem cell transplantation. Treatment studies using the mouse and dog models have shown that combining bone marrow transplantation with intra-venous gene therapy provides the best outcomes in terms of survival, behavior, and preservation of normal myelination in the central and peripheral nervous systems. With earlier diagnosis of patients through newborn screening and advances in treatment, it is hoped that more patients will have a much better quality of life.

## 1. Introduction

Krabbe disease or globoid cell leukodystrophy is an autosomal recessive disorder resulting in defective myelination in the central and peripheral nervous systems. It has an incidence of about 1 in 100,000 births in the United States and Northern Europe. The incidence can vary greatly between countries and between different ethnic groups [1]. Most of the diagnosed individuals have the infantile form with onset between 0 and 12 months, followed by a rapid downhill course, and leading to death by about two years of age when untreated. Individuals presenting in the late infantile and adult periods are also diagnosed. They currently make up about 10–20% of the patients diagnosed with Krabbe disease. Their clinical course is slower, with some surviving into the seventh decade without treatment [1]. As the birth demographics are changing in the United States, it appears that more individuals with later-onset forms are being identified. Much information regarding the incidence is being learned from the individuals identified by newborn screening.

The past 100 plus years of research on Krabbe disease has brought us from patient descriptions to the latest research on newborn screening and to the verge of new therapies for treating patients more effectively. The earliest published description of patients with globoid cell leukodystrophy was by Dr. Knud Krabbe in 1916 [2]. He described five infants who clearly showed the phenotype of infantile Krabbe disease. In 1940 [3], his name was first given to this autosomal recessive disease, although the patients in that paper may not have had Krabbe disease. The presence of the characteristic globoid cells in the white matter of the brain were described in 1924 [4]. These cells were later noted to contain a monoglycosylated ceramide and to be present in myelin [5]. In 1970, Malone [6] and Suzuki and Suzuki [7] showed that the enzyme responsible for the lysosomal hydrolysis of galactosylceramide, called galactocerebrosidase (GALC) or galactosylceramide beta-galactosidase, was deficient in patients with Krabbe disease. That was an important breakthrough, since it permitted rapid diagnosis using blood or cultured skin cells without the need for a brain biopsy. However, there was a paradox, since patients with most lysosomal disorders, like Gaucher disease, Niemann–Pick disease, and Tay–Sachs disease, store the undegraded substrate of the missing enzyme, the nervous tissues of these patients did not store large amounts of galactosylceramide. The biosynthesis of galactosylceramide is catalyzed by the enzyme UDP-galactose:ceramide galactosyltransferase (CGT), which adds galactose from uridine diphospho-galactose to ceramide and other lipid acceptors. The activity of this enzyme is highest during the period of most rapid myelination, which starts in utero in humans and at about 10 days in the mouse. The enzyme is most active in the cells making myelin, the oligodendrocytes in brain, and Schwann cells in the peripheral nervous system. CGT also catalyzes the transfer of galactose to sphingosine to make psychosine, but there is little evidence that this compound is acylated to make galactosylceramide. Psychosine may be a dead-end pathway and is hydrolyzed into galactose and sphingosine in individuals who have normal GALC activity, but not in individuals who have Krabbe disease. There is much evidence showing that psychosine is toxic when added to almost all cell types [8,9,10,11,12,13,14,15]. While the amount of psychosine produced is relatively small, there is enough to be toxic to myelinating cells, resulting in their death and a defect in myelination. In fact, in 1980, Svennerholm et al. [16] called Krabbe disease a psychosine lipidosis. More about the significance of elevated psychosine as a second-tier test in the diagnosis of Krabbe disease has recently been published [17,18].

## 2. GALC Purification and Cloning of the Gene

The purification of human GALC proved to be a difficult undertaking requiring more than 20 years of research. During that time, this laboratory attempted to purify GALC from different tissues, including placenta, liver, and brain. These tissues were extracted and subjected to many purification steps without GALC, reaching sufficient purity for protein sequencing or antibody production. After noting that the activities of other lysosomal enzymes could be measured in urine, we found we could also measure GALC activity in urine. Of course, the activity was quite low, but it was already in solution and did not require the extraction from solid tissues with thousands of other components. Through trial and error, a small amount of pure GALC was purified from 150 L of urine [19]. We found that GALC was produced as an 80 kDa precursor that was proteolytically cleaved into 30 and 50 kDa subunits. The partial amino acid sequence was obtained, and from that information, the GALC cDNA of humans was cloned [20]. Further studies led to the organization of the GALC gene [21]. Following this, the GALC cDNAs of dogs [22], twitcher mice [23], and rhesus monkeys [24] were also cloned, and the disease-causing variants in these species were identified.

## 3. Early Diagnostic Studies and Mutation Analysis

Our laboratory has always had two overlapping components: diagnosis and research. There was no physical separation between the co-workers, and findings were interchanged between each component of the laboratory. Interesting patients fueled research, and research findings aided the diagnostic testing. By 1993, before we published on the cloning of the human cDNA, we had already diagnosed over 175 patients with Krabbe disease by enzymatic testing, indicating that molecular analysis is not required for an accurate diagnosis. While most patients were infants, older patients were also diagnosed. With the sequence of the human GALC cDNA available, mutation analysis of patients with Krabbe disease could be undertaken. Initially, all of the sequencing was done manually via Sanger, and the nucleotide sequences were read from X-ray films [25]. In 1995, we first identified the 30 kb deletion as a common pathogenic variant in infantile patients with Krabbe disease [26]. At that time, we called it 502/del because the large deletion always occurred with a C>T transition polymorphism at cDNA position 502 (using the legacy numbering system). This deletion eliminates the coding region of seven exons that includes all of the 30 kDa subunit and about 15% of the coding region of the 50 kDa subunit. In this laboratory, Dr. Luzi located the 5′ and 3′ positions of the deletion, starting within the large intron 10, and going past the end of the GALC gene [27]. A rapid test for the 30 kb deletion was developed. When this mutation is found to be homozygous, the individual will always present in infancy, but when the second allele provides even a small amount of GALC activity, then the phenotype can be mild. Further studies showed that the 30 kd del was also heterozygous in some late-onset patients, including adults with Krabbe disease.

## 4. Relationship between Measured GALC Activity and Phenotype

The question of how much GALC activity is enough to either prevent the disease entirely or delay the onset until later in life is difficult to answer. The measurement of GALC activity in easily obtained tissues, such as blood or cultured skin fibroblasts, does not provide information about the activity in nervous tissues, where it is needed to maintain normal myelination. The method for measuring GALC activity using radiolabeled galactosylceramide in this laboratory has been in use since 1972. The GALC activities measured in leukocytes and cultured skin fibroblasts from infantile and late-onset patients are not statistically different from each other. However, newer methods for measuring GALC activity may be better at accurately detecting very low levels of GALC activity [28]. While there is little doubt that late-onset patients have some small amount of GALC activity, it may be difficult to measure in the tissues available for diagnostic studies. Moreover, activity measured in leukocytes and/or fibroblasts may not reflect the activity in nervous tissues where it is needed. In addition to low GALC activity, there may be a need for another insult to bring on the onset of clinical features in older patients with Krabbe disease. Insults could include an infection that affects the nervous system or immune system, a blow to the head, a hypoxic event, etc. Could some people with genetically different immune systems and APOE types be more susceptible to later-onset disease than others?

**Figure 1:**
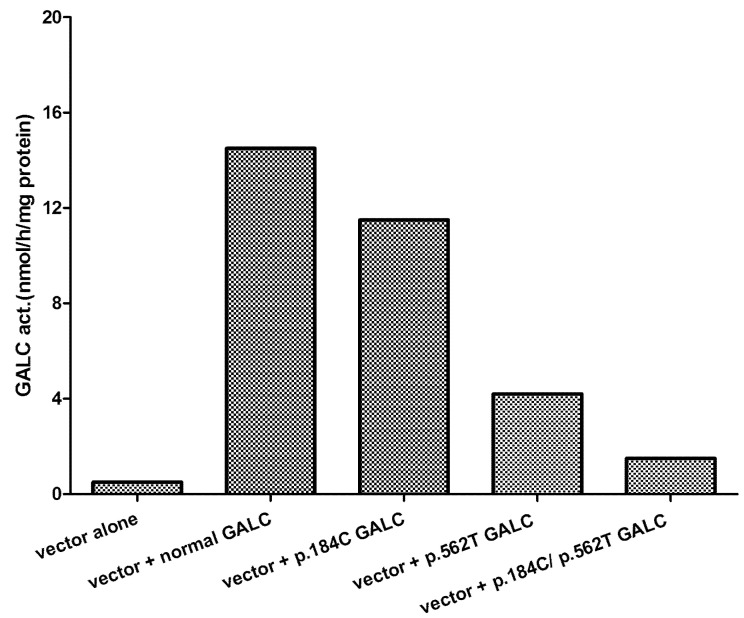
Cells of the common polymorphic variants found in the GALC gene. Cultured cells were transfected with plasmid containing either wild type GALC cDNA or GALC cDNA containing the two changes alone and together. After 72 h in culture, the cells were harvested, and GALC activity was measured.

While measurement of low GALC activity in leukocytes has been the method of choice for rapid diagnosis of Krabbe disease since 1970, it is not perfect, which became clear as more testing was being done. It is known from the earliest days of testing that there were completely normal individuals with quite low GALC activity [29]. Once the GALC gene was cloned, it became apparent that there were variants that lowered the measured GALC activity but did not cause disease even when inherited in both copies of the gene or together with a pathogenic variant. However, when some polymorphic variants are in cis with certain variants, that allele becomes disease causing [30,31]. For example, the expression of two common polymorphic variants alone and together in COS1 cells shows the effects on GALC activity (Figure 1). The presence of such polymorphisms makes carrier testing by enzyme analysis nearly impossible because both the “normal” range and “carrier” range are very wide. This has been brought home with the advent of newborn screening (NBS), which measures GALC activity in dried blood spots soon after birth [32]. As low GALC activity can be measured in dried blood spots both in individuals who will develop infantile-onset Krabbe disease and later-onset Krabbe disease, as well as individuals who have multiple copies of enzyme-lowering polymorphisms, additional testing is required to identify those infants needing immediate attention. The best method appears to be measurement of psychosine in dried blood spots or red blood cells. A dried blood spot psychosine value over 10 nmol/L indicates that the individual probably will have an infantile onset, and the parents should be counseled appropriately regarding treatment options. Values between 2 and 9 nmol/L may indicate a later-onset form. Values under 2 nmol/L indicate that the individual probably will not develop Krabbe disease. In individuals with a psychosine concentration over 2 nmol/L, mutation analysis and neuro-diagnostic studies may be indicated, but it is not a first priority [17,18].

## 5. Effect of Mutations Identified and Phenotype

What have we learned about the relationship between different mutations and the onset of clinical features? There are now over 260 variants causing Krabbe disease in addition to polymorphic changes (www.hgmd.cf.ac.uk (accessed on 30 January 2021)). Many new variants are being published from authors in Asia and other non-European countries, adding mutations to the growing list. On Table 1 and Table 2, common disease-causing variants and polymorphisms are presented. It is ideal to be able to classify a disease-causing variant as “severe” or “mild” so that when the mutations are identified in an individual, especially someone identified by NBS, a prediction can be made as to the possible type of Krabbe disease he/she will have. When a symptomatic patient is diagnosed, you already have some idea of what type of variants will be found. If an infant is symptomatic and is also homozygous for a pathogenic variant, one can surmise that the variant is severe. This is true for the 30 kb del and many other variants called “severe” in our chapter in OMMBID [1]. The 30 kb del is a common variant in patients from Europe, but is also found in patients from India and Pakistan, showing the spread of this mutation probably from Scandinavia hundreds of years ago. In our chapter on Krabbe disease in OMMBID, we have predicted that 85 variants out of 147 listed are severe, meaning that when inherited homozygous or as a compound heterozygote with another severe variant, the individuals will have early onset of disease. When two different previously unreported mutations are found in a newly diagnosed later-onset patient, it may be difficult to assign a label of severity to the alleles. However, when additional patients are identified with one of these variants together with a different variant, it may provide the information necessary to assign a severity score to the variant. It is clear is that “mild” alleles dominate “severe” alleles, meaning that even one copy of a “mild” mutation will result in a later-onset phenotype. This indicates that even a small amount of GALC activity can change the phenotype dramatically. Of course, the measured GALC activity is a contribution from each allele. While a “severe” allele may contribute little or no activity, a “mild” allele may have 2–3% of normal activity, which is enough to delay onset for many years. Therefore, inheriting two copies of a “mild” allele should provide 4–6% of normal activity, which may be enough to prevent the onset of disease for life.

Of course, variant type (missense, premature stop, large and small deletions, insertions plus insertion/deletions, splice junction changes) and location can have a wide range of effects on the GALC protein and its activity. GALC with a missense variant can have many possible effects or none. Depending on the change produced by the variant, GALC may not be processed properly in the endoplasmic reticulum (ER), the Golgi, or the lysosome [33]. If the variant causes the GALC to get misfolded and held up in the ER, it could be degraded by cellular quality-control mechanisms. If the mutation is in or near the active site, activity could be lowered because of inefficient binding of substrate or saposin activating factor. All disease-causing missense variants result in much lower GALC activity than the “normal” sequence, ranging from almost zero to 2–3% of normal. Very low activity may be difficult to accurately measure.

Variants resulting in a premature stop codon will usually result in very low GALC activity in the truncated protein. With some premature stop codons, it is possible that a small amount of mutant protein is produced. The effects of variants occurring at exon-intron splice junctions can be variable. Usually, they result in exon skipping or inclusion of some intronic sequence with unpredictable effects on the measured GALC activity. While it will probably result in very low GALC activity, there may be production of a small amount of protein, and this could lead to later-onset disease. For example, the c.195 G>C mutation occurs at an exonic splice site (before intron 1) and does not change the amino acid. Since the patients who are homozygous for this variant are all late-onset [34], there must be some active enzyme produced.

## 6. Management of Individuals Confirmed to Be at Risk for Developing Krabbe Disease

Management of individuals confirmed to have or to develop Krabbe disease will depend on their age and clinical status at the time of diagnosis. Individuals having significant neurologic deficits and clinical features at the time of diagnosis will need supportive care. However, the goal of NBS is to identify infants who will develop Krabbe disease before significant pathological damage to the nervous systems has occurred and to prepare them for treatment. Parental attitudes towards the value of NBS for Krabbe disease can be variable [35]. At this time, the standard of care in pre-symptomatic infants and mildly affected later-onset patients is hematopoietic stem cell transplantation (HSCT) [36,37]. Initially, patients were treated using blood stem cells from bone marrow; however, more recently, HSCs isolated from umbilical cord blood are being used. This greatly improves the chances of finding a suitable human leukocyte antigen match for a given patient. While this treatment has been shown to increase the lifespan of treated infantile patients, almost all present with difficulty walking and have problems with expressive language by the end of the first decade of life [38]. The walking difficulties appear to be due to a failure of HSCT to adequately correct the peripheral nervous system. Attempts to improve the protocol for treating human patients are being made using the available animal models.

## 7. Treatment of the Animal Models of Krabbe Disease

Three naturally occurring animal models of Krabbe disease with mutations in the GALC gene have been described [22,23,24]. Obviously, because of cost and ease of breeding, the twitcher (twi) mouse model has been used the most in treatment trials. These mice have one nucleotide change (p.W339X) resulting in a premature stop codon [23]. In 1984 Yeagar et al. [39] performed bone marrow transplantation (BMT) on ten-day-old twi mice, which extended their lives from 40 days to about 80 days. X-irradiation was used to prepare the mice for the BMT. They reported partial repair of the demyelination in the PNS, but no improvement of the pathology in the CNS. Both treated and untreated twi mice had tremors beginning at about post-natal day 20 (PND20). While these studies showed some extension of life and some pathological improvement with HSC treatment, it clearly was not good enough. Many other papers have been published describing other treatments, including enzyme replacement therapy, injection of neural progenitor cells, gene therapy via different routes of administration, substrate reduction therapy, chemical chaperone therapy, small molecule therapy including anti-inflammatory drugs, and combinations of these approaches [40,41,42,43,44,45,46,47,48,49,50,51,52,53,54,55,56,57,58]. These different treatments have resulted in variable degrees of success. Some of these trials only resulted in minimal extension of life and some had significant side effects and pathological events, including hepatocellular carcinoma. Other treatments involving multiple injections into the brain, BMT without myeloablation of the recipient, and use of toxic chemicals for substrate reduction probably will not be used in human patients.

At this time, studies in the mouse model indicate that AAV vectors carrying the GALC cDNA offer the best option for use in a human trial, probably in combination with the current “standard of care” HSCT [57]. While the HSCT does provide some GALC activity to the tissues, it also provides some anti-inflammatory effects to nervous tissues [41,58]. At least in twi mice, intra-venous (iv) injection of AAVrh10-mGALC alone results in normal or supra-normal GALC in all tissues, including the brain, spinal cord, and sciatic nerves, within 48 h of injection [57]. There is normal myelination in the CNS and PNS in these mice. However, combining a single iv injection of AAVrh10-GALC with BMT results in a great synergist effect with some mice living a normal lifespan [57]. The treated mice show normal myelination in the central and peripheral nervous systems. There is no evidence for neoplastic changes in the liver as reported by other laboratories using different methods of treatment. To get closer to a human trial, timing and dosing of the treatment were studied to obtain the optimum conditions [57]. Lowering the dose of AAVrh10-GALC resulted in shorter lifespans than previously published, and a higher dose of the viral vector increased the average lifespan even further, although it was not statistically significant. Delaying the injection of viral vector from one day after the BMT to five and 10 days after the BMT resulted in similar positive results. However, delaying both the BMT and the viral injection by 20 days resulted in a shorter average lifespan in the treated mice. This indicates that damage has occurred in the nervous systems that cannot be corrected. These studies help to provide a timetable for treatment, at least in the mouse model. In addition to greatly extending the lives of treated mice, there was maintenance of body weight and strength, normal myelination in the brain and sciatic nerves, and elimination of the tremor associated with twi mice. More recent studies in twi mice have shown that BMT may not be required if high dose AAVrh10-GALC is given iv early in life [59]. This treatment eliminates some of the side effects of the drugs needed before BMT.

As part of the preclinical studies, the Food and Drug Administration required toxicology studies in rats to see if this viral vector causes serious pathological changes in any tissues. Only minimal pathological changes were observed in liver and other tissues. Studies in a few dogs with Krabbe disease using BMT plus iv AAVrh10-canine GALC showed very positive results that also correlate with the dose of the viral vector [60]. While untreated affected dogs live only about 17 weeks, a 2-year-old dog treated with BMT plus high dose iv AAVrh10-canine GALC behaved completely normally and had normal myelination in the CNS and PNS. Therefore, combining HSCT, the standard of care in human patients, with an iv injection of AAVrh10-humanGALC could result in a great improvement in the quality of life for individuals treated early in their disease. Such treatment requires early diagnosis made possible by NBS and rapid confirmatory testing. At this time, two companies, Forge Biologics and Passage Bio, are starting to recruit asymptomatic and mildly symptomatic patients for gene therapy trials [see clinicaltrials.gov accessed on 30 January 2021]. With this information, families can be presented with treatment options as quickly as possible before significant pathological changes occur.

## Figures and Tables

**Table 1:** Common Pathogenic Variants in the GALC Gene.

Location	Nucleotide Change in cDNA	Codon Change	Effect	Comments *^b^*
Ex 1	c.121G>A (c.169G>A) *^a^*	GGC>AGC	p.G41S (p.G57S)	Mild, Southern Italy
Ex 4	c.284G>A (c.332G>A)	GGC>GAC	p.G95D (p.G111D)	Severe
Ex 4	c.286A>G (c.334A>G)	ACT>GCT	p.T96A (p.T112A)	Mild *^c^*
Ex 7	c.635 del + ins (c.683 del + ins)	del 12, ins3	del 5 aa + ins 2 aa	Severe, Japanese, Korean
Ex 8	c.809G>A (c.857G>A)	GGC>GAC	p.G270D (p.G286D)	Mild
Ex 8	c.860C>T (c.908C>T)	TCC>TTC	p.S287F (p.S303F)	Severe
Ex 9	c.908A>G (c.956A>G)	TAT>TGT	p.Y303C (p.Y319C)	Mild *^d^*
In10-end	30 kb deletion	30 kb del	Short mRNA	Severe
Ex 11	c.1138C>T (c.1186C>T)	CGG>TGG	p.R380W (p.R396W)	Severe
Ex 13	c.1424 delA (c.1472 delA)	TAAGG>TAGG	FS, PS	Severe
Ex 14	c.1538 C>T (c.1586C>T)	ACG>ATG	p.T513M (p.T529M)	Severe
Ex 15	c.1652A>C (c.1700A>C)	TAC>TCC	p.Y551S (p.Y567S)	Severe
Ex 16	c.1853T>C) (c.1901T>C)	TTA>TCA	p.L618S (p.L634S)	Mild (Asian)

*^a^* Location of variants and protein changes in the original numbering system are shown first; those in parentheses are based on a start codon 48 nucleotides longer. *^b^* The comments reflect the best information available from published and unpublished data. *^c^* May only be disease causing when the p.I562T polymorphism is present on the same allele. *^d^* May only be disease causing when the other allele is considered “severe” or when a polymorphism is present on the same allele. Note: Many disease-causing mutations found in the GALC gene also contain polymorphisms in the same copy of the gene. Abbreviations: aa = amino acids; del = deletion; ins = insertion; c. = cDNA; p. = protein; FS = frame shift; PS = premature stop.

**Table 2:** Common Polymorphic Variants in the GALC gene.

Location	Nucleotide Change in cDNA	Codon Change	Effect	Comments *^b^*
Ex 4	c.502C>T (c.550C>T) *^a^*	CGT>TGT	p.R168C (p.R184C)	The 30 kb deletion always has this polymorphism
Ex 6	c.694G>A (c.742G>A)	GAT>AAT	p.D232N (p.D248N)	
Ex 8	c.865A>G (c.913A>G)	ATC>GTC	p.I289V (p.I305V)	Japanese
Ex 14	c.1637T>C (c.1685T>C)	ATA>ACA	p.I546T (p.I562T)	Very common

*^a^* Location of mutations and protein changes in the original numbering system are shown first (legacy); those in parentheses are based on a start codon 48 nucleotides longer. *^b^* The comments reflect the best information available from published and unpublished data.
